# 2017 WSES guidelines for the management of iatrogenic colonoscopy perforation

**DOI:** 10.1186/s13017-018-0162-9

**Published:** 2018-01-24

**Authors:** Nicola de’Angelis, Salomone Di Saverio, Osvaldo Chiara, Massimo Sartelli, Aleix Martínez-Pérez, Franca Patrizi, Dieter G. Weber, Luca Ansaloni, Walter Biffl, Offir Ben-Ishay, Miklosh Bala, Francesco Brunetti, Federica Gaiani, Solafah Abdalla, Aurelien Amiot, Hany Bahouth, Giorgio Bianchi, Daniel Casanova, Federico Coccolini, Raul Coimbra, Gian Luigi de’Angelis, Belinda De Simone, Gustavo P. Fraga, Pietro Genova, Rao Ivatury, Jeffry L. Kashuk, Andrew W. Kirkpatrick, Yann Le Baleur, Fernando Machado, Gustavo M. Machain, Ronald V. Maier, Alain Chichom-Mefire, Riccardo Memeo, Carlos Mesquita, Juan Carlos Salamea Molina, Massimiliano Mutignani, Ramiro Manzano-Núñez, Carlos Ordoñez, Andrew B. Peitzman, Bruno M. Pereira, Edoardo Picetti, Michele Pisano, Juan Carlos Puyana, Sandro Rizoli, Mohammed Siddiqui, Iradj Sobhani, Richard P. ten Broek, Luigi Zorcolo, Maria Clotilde Carra, Yoram Kluger, Fausto Catena

**Affiliations:** 1 Unit of Digestive, Hepato-Pancreato-Biliary Surgery and Liver Transplantation, Henri Mondor University Hospital, AP-HP, and University of Paris Est, UPEC, 51 Avenue du Maréchal de Lattre de Tassigny, 94010 Créteil, France; 2 Department of Surgery, Maggiore Hospital, Bologna, Italy; 3 General Surgery and Trauma Team, Niguarda Hospital, Milan, Italy; 4 Department of Surgery, Macerata Hospital, Macerata, Italy; 5 Department of General and Digestive Surgery, University Hospital Dr Peset, Valencia, Spain; 6 Unit of Gastroenterology and Endoscopy, Maggiore Hospital, Bologna, Italy; 7 Department of Trauma Surgery, Royal Perth Hospital, Perth, Australia; 8 General Surgery I, Papa Giovanni XXIII Hospital, Bergamo, Italy; 9 Acute Care Surgery at The Queen’s Medical Center, John A. Burns School of Medicine, University of Hawaii, Honolulu, USA; 10 Department of General Surgery, Rambam Healthcare Campus, Haifa, Israel; 11 Trauma and Acute Care Surgery Unit, Hadassah Hebrew University Medical Center, Jerusalem, Israel; 12 Gastroenterology and Endoscopy Unit, University Hospital of Parma, Parma, Italy; 13 Department of Gastroenterology and Digestive Endoscopy, Henri Mondor Hospital, AP-HP, and University of Paris Est, UPEC, Creteil, France; 14 Unit of Digestive Surgery and Liver Transplantation, University Hospital Marqués de Valdecilla, University of Cantabria, Santander, Spain; 15 Department of Surgery, UC San Diego Health System, San Diego, CA USA; 16 Unit of Digestive Surgery, Cannes Hospital, Cannes, France; 17 Division of Trauma Surgery, Department of Surgery, School of Medical Sciences, University of Campinas (Unicamp), Campinas, SP Brazil; 18 Department of General and Oncological Surgery, University Hospital Paolo Giaccone, Palermo, Italy; 19 Virginia Commonwealth University, Richmond, VA USA; 20 Assia Medical Group, Department of Surgery, Sackler School of Medicine, Tel Aviv University, Tel Aviv, Israel; 21 Department of Surgery, Critical Care Medicine and the Regional Trauma Service, Foothills Medical Center, Calgari, AB Canada; 22 Department of Emergency Surgery, Hospital de Clínicas, School of Medicine, UDELAR, Montevideo, Uruguay; 23 Il Cátedra de Clínica Quirúgica, Hospital de Clínicas, Facultad de Ciencias Medicas, Universidad National de Asuncion, Asuncion, Paraguay; 24 Department of Surgery, University of Washington, Seattle, WA USA; 25 Department of Surgery and Obstetrics/Gynecologic, Regional Hospital, Limbe, Cameroon; 26 Unit of General Surgery and Liver Transplantation, Policlinico di Bari “M. Rubino”, Bari, Italy; 27 Unit of General and Emergency Surgery, Trauma Center, Centro Hospitalar e Universitario de Coimbra, Coimbra, Portugal; 28 Department of Trauma and Emergency Center, Vicente Corral Moscoso Hospital, University of Azuay, Cuenca, Ecuador; 29 Digestive and Interventional Endoscopy Unit, Niguarda Ca’Granda Hospital, Milan, Italy; 30 Department of Surgery and Critical Care, Universidad del Valle, Fundacion Valle del Lili, Cali, Colombia; 31 Department of Surgery, UPMC, University of Pittsburg, School of Medicine, Pittsburg, USA; 32 Department of Anesthesiology and Intensive Care, University Hospital of Parma, Parma, Italy; 33 Critical Care Medicine, University of Pittsburg, School of Medicine, Pittsburg, USA; 34 Trauma and Acute Care Service, St Michael’s Hospital, Toronto, ON Canada; 35 Department of Surgery, Radboud University Medical Center, Nijmegen, The Netherlands; 36 Department of Surgery, Colorectal Surgery Unit, University of Cagliari, Cagliari, Italy; 37 University Paris Diderot, Rothschild Hospital, AP-HP, Paris, France; 38 Department of Emergency and Trauma Surgery of the University Hospital of Parma, Parma, Italy

**Keywords:** Iatrogenic colonoscopy perforation, Colonoscopy, Gastrointestinal endoscopy, Emergency surgery, Laparoscopy, Antibiotic therapy, Intra-abdominal infection, Open abdomen

## Abstract

Iatrogenic colonoscopy perforation (ICP) is a severe complication that can occur during both diagnostic and therapeutic procedures. Although 45–60% of ICPs are diagnosed by the endoscopist while performing the colonoscopy, many ICPs are not immediately recognized but are instead suspected on the basis of clinical signs and symptoms that occur after the endoscopic procedure. There are three main therapeutic options for ICPs: endoscopic repair, conservative therapy, and surgery. The therapeutic approach must vary based on the setting of the diagnosis (intra- or post-colonoscopy), the type of ICP, the characteristics and general status of the patient, the operator’s level of experience, and surgical device availability.

Although ICPs have been the focus of numerous publications, no guidelines have been created to standardize the management of ICPs. The aim of this article is to present the World Society of Emergency Surgery (WSES) guidelines for the management of ICP, which are intended to be used as a tool to promote global standards of care in case of ICP. These guidelines are not meant to substitute providers’ clinical judgment for individual patients, and they may need to be modified based on the medical team’s level of experience and the availability of local resources.

## Background

Iatrogenic colonic perforations (ICPs) are an infrequent but severe complication of colonoscopy. Globally, the incidence is estimated to be 0.016–0.8% for diagnostic colonoscopies and 0.02–8% for therapeutic colonoscopies [1–10], but considering the increasing numbers of screening, diagnostic, and therapeutic colonoscopies being performed every year, the frequency of ICP is not insignificant [11, 12].

Approximately 45–60% of ICPs are detected by the endoscopist while performing the colonoscopy, although a considerable number of ICPs are not recognized immediately, but rather are suspected on the basis of clinical signs and symptoms occurring after the endoscopic procedure. In this latter case, colonic perforations may lead to the development of secondary peritonitis, which is associated with significant morbidity and mortality [5, 13–18]. Depending on the delay in the management of the ICP and the pre-existing pathologies, ICP-related mortality is as high as 5–25% [5, 14–16, 18–22].

One of the most important issues in the management of ICPs is the time period between the diagnosis and the treatment. There are different treatment alternatives for ICP, including conservative, endoscopic, and surgical approaches. The therapeutic strategy varies based on the setting in which the ICP is diagnosed (i.e., intra- or post-colonoscopy), the specific characteristics of the perforation (e.g., size, location, and etiology), the patient’s general status, and the skill level of the operator [8, 23, 24]. Although ICPs have been the subject of numerous publications, no randomized clinical trials have been conducted to evaluate the best treatment option and no guidelines have been defined to standardize its management. For this reason, the World Society of Emergency Surgery (WSES) convened a consensus conference to review the available literature, discuss the current controversies, and create guidelines for the management of ICP. The present article is the summary of the WSES consensus conference, including (1) the incidence of and risk factors for ICP, (2) the diagnosis of ICP, (3) the conservative and endoscopic treatments for ICP, (4) the surgical treatments for ICP, and (5) the follow-up after ICP treatment. Based upon the evidence emerging from the consensus conference, a decision-making algorithm was developed to guide clinicians and surgeons through the different medical, endoscopic, and surgical treatments for ICP.

## Materials and methods: expert panel and consensus conference organization

On September 2016, the President of the WSES (Luca Ansaloni) appointed five WSES members (Nicola de’Angelis, Fausto Catena, Federico Coccolini, Salomone Di Saverio, Massimo Sartelli) to establish the project committee and determine the organization of an international multidisciplinary expert panel deputed to develop the WSES Guidelines for the management of ICP. The project committee agreed to develop practice guidelines by formal consensus, which consists of formalizing the degree of agreement among experts by identifying and selecting, through ratings and feedback, the points on which the experts agree and the points on which they disagree or are undecided. Additionally, it involves drafting a small number of concise and unambiguous recommendations that address the questions asked.

Briefly, the development of the WSES guidelines was structured upon two phases: the synthesis of the literature and the consensus conference. For phase I, the project committee identified 17 key questions regarding ICP risk, diagnosis, and treatments that would guide the literature search (Table 1). Then, an expert panel composed of surgeons, endoscopists, gastroenterologists, and anesthesiologists from five continents was invited to participate and answer the selected questions. The experts who agreed to participate (*n* = 50) were divided into 17 groups of at least 3 experts each who were asked to answer one of the selected key questions regarding ICP. For each group, a group leader was nominated; the group leader was responsible for coordinating the work of the experts in his/her group, providing a summary document that aligned the group’s agreement upon answers to the specific question assigned, and meeting the assigned deadline. Experts were solicited to search the literature using a systematic approach within different databases (e.g., PubMed, EMBASE, and Scopus) and assess the level of evidence and the grade of the recommendation based on the recommendations of Guyatt et al. [25] (Table 2). For the literature search, the following keywords and MeSH terms were used: management of colonic/colon perforations, repair of iatrogenic large bowel perforations, abdominal imaging in colonic perforations, evolution of imaging, colonic perforation complications/outcomes, endoscopic treatment of colonic perforations, and peritonitis after colonoscopy.

**Table 1:** Key questions used to develop the Consensus Conference on iatrogenic colonoscopy perforation (ICP)

Risk of ICP	
Q1	What are the general recommendations to minimize the risk of ICP during screening and therapeutic colonoscopies?
Q2	What is the maximum incidence of ICP considered acceptable for centers where diagnostic or therapeutic colonoscopies are performed?
Diagnosis of ICP	
Q3	What is the minimum information the endoscopist must report after diagnosing an ICP during a colonoscopy procedure?
Q4	What are the minimum biochemical and imaging investigations that should be requested in the case of suspected ICP?
Conservative and endoscopic treatments of ICP	
Q5	What are the indications for a conservative treatment or an immediate surgical intervention after an ICP diagnosis?
Q6	What is the minimum duration of the hospital observation period for patients who have undergone successful endoscopic closure or conservative management of ICP?
Q7	What investigations (clinical, biochemical, and imaging) should be performed during the observation period in patients who have undergone successful endoscopic closure or conservative management of ICP?
Q8	What is the recommended type and duration of antibiotic therapy in patients who have undergone successful endoscopic closure or conservative management of ICP?
Q9	What is the recommended type and duration of antithrombotic prophylaxis in patients who have undergone successful endoscopic closure or conservative management of ICP?
Q10	How long is the fasting time in patients who have undergone successful endoscopic closure or conservative treatments for ICP?
Surgical treatment of ICP	
Q11	Is explorative laparoscopy indicated in all patients with ICP?
Q12	What are the indications for conversion from laparoscopy to open surgery in patients with surgical ICP?
Q13	What are the key factors when choosing the best surgical approach for ICP?
Q14	What are the indications for performing a diverting or terminal stoma in patients with ICP?
Q15	What are the indications for drainages in patients with ICP?
Q16	What are the indications for the use of damage control surgery in patients with ICP?
Follow-up of ICP	
Q17	Is there any recommendation to perform a surveillance endoscopy after a successful ICP treatment? If so, what is the recommended timing for it?

**Table 2:** Grading of recommendations (from Guyatt et al.)

Grade of recommendation	Description	Benefits vs. risks	Quality of supporting evidence	Implications
1A	Strong recommendation, high-quality evidence	Benefits clearly outweigh risks and burdens, or vice versa	RCTs without important limitations or overwhelming evidence from observational studies	Strong recommendation, applies to most patients in most circumstances without reservation
1B	Strong recommendation, moderate-quality evidence	Benefits clearly outweigh risk and burdens, or vice versa	RCTs with important limitations (inconsistent results, methodological flaws, indirect or imprecise) or exceptionally strong evidence from observational studies	Strong recommendation, applies to most patients in most circumstances without reservation
1C	Strong recommendation, low-quality or very low-quality evidence	Benefits clearly outweigh risk and burdens, or vice versa	Observational studies or case series	Strong recommendation based on limited evidence; recommendations may change when higher quality or more extensive evidence becomes available
2A	Weak recommendation, high-quality evidence	Benefits closely balanced with risks and burdens	RCTs without important limitations or overwhelming evidence from observational studies	Weak recommendation; best action may differ depending on circumstances, expertise of clinician, the patient in question, or other social issues
2B	Weak recommendation, moderate-quality evidence	Benefits closely balanced with risks and burdens	RCTs with important limitations (inconsistent results, methodological flaws, indirect or imprecise) or exceptionally strong evidence from observational studies	Weak recommendation; best action may differ depending on circumstances, expertise of clinician, the patient in question, or other social issues
2C	Weak recommendation, low-quality or very low quality evidence	Uncertainty in the estimates of benefits, risks, and burdens; benefits, risks, and burdens may be closely balanced	Observational studies or case series	Very weak recommendation; other alternatives may be equally reasonable

Within each group, a scientific discussion ensued via email, and modifications were implemented when necessary based on feedback, consistent evidence from the literature, and, whenever pertinent, clinical experience (empirical evidence). The answers provided for each question constituted the provisional statements about the management of ICP that were submitted for review to all participants at the consensus conference (phase II). The Consensus Conference on ICP management was held in Campinas, Brazil, on May 20, 2017, during the 4th WSES World Congress. During the first part of the consensus conference, the group leaders presented the results of their group discussion with the answer to the key question assigned, the provisional statements along with the supporting literature, the level of evidence, and the grade of the recommendation. Each statement was then discussed and voted on by the audience. The percentage of agreement was recorded immediately; in cases of disagreement greater than 30%, the statement was modified after discussion. Furthermore, relevant comments about each statement were collected and used during the revision process. During the final portion of the consensus conference, a comprehensive algorithm for the management of ICP was developed based on the results of the literature review and the plenary discussion among the experts.

The revised statements, their level of evidence, and the recommendation grade are presented below. Please note that the WSES guidelines must be considered an adjunctive tool in the decision-making process regarding the management of ICP; they are not intended to substitute a provider’s clinical judgment for an individual patient, and they may need to be modified based on the medical team’s experience and the available local resources.

## Results

### Incidence of and risk factors for ICP

#### What are the general recommendations for minimizing the risk of ICP during screening and therapeutic colonoscopies?

There are a number of risk factors that have been related to ICP in the literature (Table 3). Older patients are more vulnerable to ICP, and the ages of 65, 75, and 80 years have been shown to be independent risk factors for ICPs [23, 26, 27]. Female gender [28, 29], low BMI [28, 30], low albumin level, the presence of comorbidities, diverticulosis, Crohn’s disease, and admission to an ICU are also acknowledged to be risk factors in several studies [20, 23, 26, 28]. The endoscopist’s level of experience may also be considered a risk indicator, as higher incidences of ICP have been reported for non-gastroenterologist endoscopists and low-volume endoscopy centers [31–33]. Finally, anesthesia during colonoscopy has been associated with an increased risk of ICP, in relation to the worsening of patient’s comorbidities and the increasing technical complexity of these procedures [34, 35].

**Table 3:** Principal risk factors for iatrogenic colonoscopy perforations (ICP)

Risk factors	References
Increasing age (> 65 years)	[18, 23, 26, 27, 36]
Female gender	[18, 28, 29, 36]
Low BMI	[28, 29]
Low albumin level	[20, 23, 26, 28]
Presence of comorbidities	[18, 36]
Crohn’s disease and diverticulosis	[16, 18, 20, 23, 26, 28]
Admission in ICU	[20, 23, 26, 28]
Endoscopist’s experience	[18, 29, 31–33]
Non-gastroenterologist endoscopists	[31, 33]
Low volume centers	[31, 33]
Previous abdominal surgery	[16, 36]
Colonic obstruction	[16, 18]
Bevacizumab therapy	[44, 46, 47]
Therapeutic vs. diagnostic procedure	[5, 10, 37–42, 44, 49]
Colonoscopy vs. sigmoidoscopy	[5, 29, 36]
General anesthesia	[34, 35]

In a recent study of 56,882 colonoscopies, full-thickness large bowel perforation occurred in forty patients, corresponding to an incidence rate of 0.07% (0.05% in diagnostic/screening procedures and 0.17% in therapeutic colonoscopies) [18]. A greater risk of ICP was associated with low-volume practices, female gender (due to greater colonic length and a more mobile transverse colon), advanced age (reduced wall strength), history of diverticular disease, previous abdominal surgery (especially pelvic), and colonic obstruction (risk of over-insufflation).

In a Spanish study of 16,285 colonoscopies, ICPs were reported in 0.09% of cases [16]. Colonic obstruction, prior abdominal surgery, and sigmoid diverticular disease were indicated as potential risk factors.

A review from the Netherlands including 30,366 endoscopic procedures found that ICP occurred in 35 patients (0.12%) [5]. The authors described a 4-fold higher risk of ICP in colonoscopies compared with sigmoidoscopies and a 5-fold greater risk of ICP in therapeutic compared with diagnostic procedures.

A review of 10,486 colonoscopies performed in a single institution included 20 ICPs over a period of 10 years (corresponding to an incidence rate of 0.19%) [29]. During the same time interval, 46,501 flexible sigmoidoscopies were performed and only two ICPs occurred (0.004%). Female patients had significantly more ICPs compared with males and, although not statistically significant, the risk of ICP was numerically higher for endoscopists in training than experienced endoscopists [29].

In a review of studies published between 2001 and 2009 analyzing 969,913 colonoscopies [36], the incidence of ICP ranged from 0.032 to 0.14%. The risk factors for ICP included age over 75 years (4- to 6-fold increase), colonoscopy instead of sigmoidoscopy (2–4 times greater), female gender, diverticular disease, previous abdominal surgery, and multiple comorbidities, including diabetes mellitus, chronic pulmonary disease, congestive heart failure, myocardial infarction, cerebrovascular disease, peripheral vascular disease, renal insufficiency, liver disease, and dementia.

Therapeutic colonoscopies generally involved a higher risk for ICP, particularly the following procedures: polypectomy for large polyps, multiple polypectomies, pneumatic dilatation for Crohn’s stricture [37], the use of argon plasma coagulation, and endoscopic mucosal resection (EMR) and endoscopic submucosal dissection (ESD) for colorectal neoplasia [38]. For endoscopic polypectomies, the related perforation risk has been related to the size of the polyp (larger than 10 mm in the right colon or 20 mm in the left colon) and a sessile morphology [38], and it is considered to be less than 1%, even when more challenging polypectomy techniques such as EMR are performed [39]. Complex procedures such as EMR and ESD are associated with a higher perforation incidence and should be considered to have a high risk of ICP. In 2014, a meta-analysis by Wang et al. comparing procedure-related complications in EMR and ESD for colorectal tumors (including 4 retrospective case-control studies) reported ESD-related perforations in 31/347 cases and EMR-related perforations in 33/566 cases [40]. The current literature demonstrates that the perforation risk for ESD is decreasing in higher volume centers to less than 5% [41, 42].

Perforation in colorectal stenting is the main early adverse event [43]. Use of a self-expandable metal stent (SEMS) has been associated with an overall perforation rate of 7–8% [10, 44]. In cases of acute malignant colonic obstruction, retrospective studies have shown an SEMS-related perforation risk of 5–9% [45]. Stenting of either benign or neoplastic strictures has been associated with a 7.4% incidence of ICP in a recent meta-analysis [43]; the type of stent, benign etiology, bevacizumab therapy, and the need for re-dilation have been identified as risk factors for ICP [44, 46, 47].

Endoscopic balloon dilation may entail perforation rates up to 11%, even though the rate of iatrogenic perforation for Crohn’s disease stricture treatment is less than 5% in the majority of retrospective studies [37, 45, 48]. Balloon dilation of rectal anastomotic strictures has been associated with a 1.1% rate of ICP [49].

**Fig. 1:**
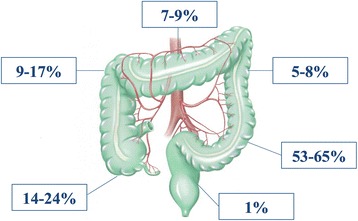
Location and frequency of iatrogenic colonoscopy perforation

The most common site of perforation is the sigmoid colon (53–65%), followed by the cecum, the ascending colon, the transverse colon, the descending colon, and the rectum [6, 13, 15, 29, 50] (Fig. 1). ICPs are generally intra-peritoneal perforations; extra-peritoneal perforations may manifest as pneumoretroperitoneum, pneumomediastinum, or subcutaneous emphysema. Combined intra- and extra-peritoneal perforations have been reported anecdotally [51].

There is only one randomized study concerning the risk factors and preventive measures for ICP, whereas several reviews of large clinical series and meta-analyses to define the incidence and risk factors for ICP have been published [52, 53]. Recommendations for preventive measures derive from these studies and expert opinions [54].

##### Statement 1

1.1. * During diagnostic endoscopy training, a low threshold at which the senior endoscopist should assume manual control or abort the procedure should be established. Unusual difficulty in traversing the sigmoid colon, a difficult examination in a female or elderly patient, or the presence of diverticular disease or colonic obstruction should be considered alarming conditions (Recommendation Grade 1C).*
1.2. * During diagnostic or screening colonoscopies, endoscope progression should be gently performed and loop formation avoided. Alternative maneuvers (*e.g.*, compression, decubitus changes) should be used in case of pain, but when difficulties in the progression are observed, it is recommended to abort the procedure (Recommendation Grade 1C).*
1.3. * Air should be insufflated judiciously to avoid barotrauma, especially if bowel obstruction is suspected. The use of CO*_*2*_ *further minimizes bowel distension, abdominal discomfort, and the risk of perforation (Recommendation Grade 1B).*
1.4. * During en bloc endoscopic polypectomy, the maximum size of the tissue sample safely included in the SNARE should be 2 cm (especially if the lesion is proximal to the splenic flexure). Pre-polypectomy submucosal injection reduces the risk of electro-coagulative damage to the muscularis propria. Blended current mode limits the depth of tissue damage, and cold techniques are preferred for small polyps (≤5 mm) (Recommendation Grade 1C).*
1.5. * Endoscopic submucosal dissection (ESD) should be limited to selected cases because of the high rate of associated complications (Recommendation Grade 1C).*
1.6. * Stenting of a malignant disease should be discouraged in patients receiving bevacizumab. In the case of Crohn’s disease, dilatation of a long stenotic area in the presence of active disease or a suspected fistula before or after stent placement is not advisable (Recommendation Grade 1C).*
1.7. * Whenever risky endoscopic procedures must be performed, the availability of and close collaboration with a hospital-based multidisciplinary team can improve patient outcomes (Recommendation Grade 1C).*

#### What is the maximum incidence of ICP considered acceptable for centers where diagnostic or therapeutic colonoscopies are performed?

Colonoscopy has been demonstrated to be the most cost-effective method for colorectal cancer screening. As the number of procedures performed worldwide is increasing, gastrointestinal professional societies have adopted strict safety standards for endoscopic practice, including the monitoring and auditing of complications to detect performance gaps and continuously improve the safety of colonoscopy [55]. The American Society for Gastrointestinal Endoscopy (ASGE)/American College of Gastroenterology (ACG) Task Force on Quality in Endoscopy recommends that post-colonoscopy perforation rates should be maintained at ≤ 1 per 500 colonoscopies (≤ 1/1000 in screening healthy subjects) [56]. For screening colonoscopies, the European Society of Gastrointestinal Endoscopy (ESGE) proposes that perforation should require surgery in ≤ 1/1000 [57]. In an audit of post-colonoscopy complications before starting national colorectal cancer screening, the British Society of Gastroenterology (BSG) reported post-colonoscopy perforation rates of 1/769 over a total of 9223 colonoscopies [58].

##### Statement 2

2.1. * The maximum acceptable incidence of ICP for diagnostic colonoscopies should not exceed 0.1% (Recommendation Grade 1A).*
2.2. * During therapeutic colonoscopy, the maximum acceptable incidence of ICP should be ≤ 1% for complex polypectomy (Recommendation 1A) and less than 7% for SEMS placement (Recommendation Grade 1C).*

### Diagnosis of ICP

#### What is the minimum information the endoscopist must report after diagnosing an ICP during a colonoscopy procedure?

Perforation during diagnostic or screening endoscopic procedures may occur from one of these two main pathways: (a) direct mechanical damage to the colonic wall by the tip or the side of the endoscope as it is pushed forward or (b) a pneumatic distension due to barotrauma (Table 4). Direct mechanical trauma is the most frequent etiology of ICP, and perforations originating from mechanical trauma are commonly large and located in the sigmoid region. The injury is usually produced by direct trauma due to an inaccurate instrumental insertion, colonoscope movements toward the mucosal surface, retro-flexion maneuvers, or excessive torsion. Indirect injuries can also occur as the consequence of bowing or stretching the distal part of the colon. The presence of redundant colon diverticula or adhesions from previous surgeries can increase the risk of mechanical trauma during colonoscopy [16]. Barotrauma is instead produced by the excessive distension of the bowel due to over-insufflation, which produces linear lacerations at the colonic wall that may evolve into full-thickness defects. This type of perforation is more frequently located at the cecal region, where the thinner muscular layer and the larger lumen diameter make this region more vulnerable to pressure-related injuries [6, 16, 59, 60]. For interventional endoscopies, the mechanism of perforation can be the same as those occurring during diagnostic endoscopy, or they may be due to thermal/electrical injury of the colonic wall, manifesting as a wall ischemia. In this latter case, the perforation can occur with a delay of 24–72 h [18, 54]. Wall damage can be incomplete and the perforation concealed as it is confined by the surrounding tissues. During the following days or weeks, an abscess may develop that may delay the diagnosis.

**Table 4:** Main etiologies of iatrogenic colonoscopy perforation (ICP)

Type of injury	
• Direct mechanical trauma	
• Barotrauma	
• Thermal/electrical injury	
Endoscopic therapeutic procedures at risk for ICP	
• Colorectal stenting	
• Polypectomy	
• Colonic dilation	
• Argon plasma coagulation (APC)	
• Endoscopic mucosal resection (EMR)	
• Endoscopic submucosal dissection (ESD)	

Up to 60% of ICPs are detected by the endoscopist while performing the procedure [14, 16, 18, 60–62]. In a retrospective evaluation of a single institution, 68% of ICPs were identified on the day of endoscopy, 23% on day 1 or 2 after the endoscopy, and 9% were identified at least 2 weeks after the procedure [29]. The results of a survey of 30,336 colonoscopies showed a mean delay of 0.36 days for the diagnosis of ICP after diagnostic endoscopies and 1.5 days after therapeutic procedures [5].

##### Statement 3

3.1. * If the ICP is detected during the procedure by the endoscopist, a detailed description should be provided including the following information*:
*Colonoscopy indication (i.e., diagnostic or therapeutic)*
*Associated colonic pathology (e.g., strictures, polyps, tumors)*
*Administration of sedation, analgesia, or anesthesia for the colonoscopy*
*Patient’s general status and presence of comorbidities*
*Gas type used for insufflation*
*Quality of the colonic preparation*
*Time of the ICP occurrence*
  *Most likely reason for ICP (*e.g.*, thermal injury, mechanical injury)*
*Injury localization and size*
*Whether an endoscopic resolution was intended, attempted, or completed*
*How the endoscopic repair was performed*
*Presence of abdominal distention increasing the probability of abdominal compartment syndrome*
  *This recommendation was obtained by consensus after discussion with the panel experts (Recommendation Grade 2C)*.

#### Which are the minimum biochemical and imaging investigations that should be requested in the case of a suspected ICP?

A delay in the diagnosis of ICP is a critical issue for therapeutic outcomes; when the diagnosis is delayed more than 24 h, the chance increases that more invasive treatments (e.g., surgery) will be required [2, 63]. Physicians should therefore search for this potentially life-threatening complication and run clinical and biochemical tests if an ICP is suspected.

An ICP can be appreciated by direct visualization of the parietal defect or the view of intra-abdominal tissues through the colonic wall during the endoscopy [15]. Otherwise, the diagnosis of ICP is based on clinical, laboratory, and radiologic findings [64]. The clinical presentation of an ICP can vary widely, depending on the size of the perforation, the type of etiologic agent, the affected colonic location, the degree of intra-peritoneal contamination, and the patient’s general status. In the majority of patients (91–92%), symptoms develop within the first 48 h following the completion of the endoscopy [14, 29]. The most common symptom is abdominal pain associated with distension, although painless cases of ICP or cases with severe cramp-like pain have been described [13, 16, 18]. In two large clinical series, the most consistent symptoms were abdominal pain (from 74 to 95%), guarding/rebound tenderness (82.5) with diffuse peritonitis, tachycardia (62.5%), leukocytosis (40%), fever (38%), rectal bleeding (15%), and isolated abdominal distension (6.6%) [16, 18]. Only a small number of patients with ICP (5%) remained asymptomatic [52, 59]. An unusual clinical sign (1/55 patients with ICPs) was a delayed subcutaneous emphysema and an ongoing necrotizing infection of the abdominal wall [16, 18]. It is a common belief that patients with diffuse peritonitis can be diagnosed and treated for perforation on a clinical basis, but peritonitis-like clinical scenarios can also occur in the absence of perforation. For instance, a transmural thermal injury after polypectomy with serosal irritation without any obvious perforation produces localized peritonitis that is amenable to non-operative management. Thus, biochemical and imaging studies are always indicated when an ICP is suspected.

Laboratory tests should be run for inflammatory markers that can reveal severe bacterial infections associated with the perforation [65], such as white blood cell count (WBC) and C-reactive protein (CRP) [66, 67]. In case of delayed presentation (> 12 h), the pro-calcitonin level (PCT) can be useful for ICP diagnosis.

Perforations of intra-peritoneal segments of the colon (e.g., the cecum, transverse colon, or sigmoid colon) more often lead to free intra-peritoneal fluid and air (large amounts in cases of barotrauma from insufflation), whereas perforations of the ascending and descending colon and rectum or wall injuries contained in the supplying mesentery result mainly in extra-peritoneal air. Mixed situations are possible if the perforation is in the middle between an intra- and extra-peritoneal portion [68]. Upright or decubitus abdominal radiographs can detect small amounts of free peritoneal air, but they are insensitive to the presence of fluid. Plain thoracic and abdominal radiographs have a positive predictive value (PPV) of 92% for ICPs [13]. Of note, the PPV has been shown to be higher for ICPs occurring during diagnostic procedures (PPV 100%) than for ICPs occurring during therapeutic procedures (PPV 45%) [2]. Alternatively, an ultrasound may be useful in cases in which the radiation burden should be limited, notably in children and pregnant women. However, this method should not be considered definitive in excluding a pneumoperitoneum [69].

If the clinical suspicion of ICP persists after a normal plain radiograph, a computed tomography (CT) scan with contrast enhancement should be requested, as this imaging tool can easily detect small amounts of both free intra-peritoneal air and fluids, in some cases with the foci of the gas congregating near the perforation site [68]. Air trapped in the mesenteric folds is found in perforation of the colon. A pneumoretroperitoneum is caused by extraperitoneal perforations such as perforations of the descending colon and rectum. Gas in the right anterior pararenal space indicates ascending colon perforation, whereas gas in the left pararenal space indicates descending or sigmoid colon perforations. Generally, rectal perforation causes bilateral pneumoretroperitoneum [70]. For extra-peritoneal perforations, the CT scan can show air tracking along the mesenteric and fascial planes, even in the mediastinum and abdominal, and chest and neck walls. Of note, the retro-peritoneal air dissecting the mediastinum and the retropharyngeal tissues can cause a change in the tone of the larynx, resulting in voice change [71].

Colonoscopy may also dissect within the wall of the colon with pneumatosis. Moreover, mucosal injury and intraluminal pressure may dissect air inside the mesenteric and portal venous system. For all these reasons, CT is much more effective in the diagnosis of extraluminal air compared to conventional radiography [15]. Double contrast CT (intravenous and rectal) is increasingly used in patients with clinical suspicion of ICP and without diffuse peritonitis. This diagnostic tool may be useful for detecting concealed or sealed perforations that are eligible for non-operative management [72]. Multi-detector CT (MDCT) is superior to single helical or conventional CT because it can provide rapid, high-volume coverage, and diagnostic images, even in patients who are unable to perform prolonged breath holds. One study showed that MDCT was 86% accurate in predicting the site of perforation [69].

The following recommendations were developed using a large clinical series and expert opinions, since randomized studies on this topic are lacking.

##### Statement 4

4.1. * After diagnostic or therapeutic colonoscopies, all patients who present with abdominal pain, and/or tenderness, and/or abdominal distension, and/or fever, and/or rectal bleeding should be investigated for ICP by laboratory tests and imaging exams (Recommendation Grade 1B).*
4.2. * The minimum biochemical markers that should be requested in the case of suspected ICP are white blood cell count and C-reactive protein (Recommendation Grade 1C).*
4.3. * ICP should be confirmed with the demonstration of free intra-peritoneal or extra-peritoneal air (Recommendation 1B). CT scan is more sensitive than standard abdominal radiographs to detect free air (Recommendation Grade 1C).*
4.4. * In the case of localized peritoneal signs, double contrast enhanced CT scan can be a useful adjunctive tool to confirm the feasibility of non-operative management of ICP (Recommendation Grade 1C).*

### Conservative and endoscopic treatments for ICP

#### Which are the indications for conservative treatment or an immediate surgical intervention after an ICP diagnosis?

Once the diagnosis of perforation is confirmed by clinical and radiological examinations, the decision between surgical and non-operative treatments will depend on the type of injury, the quality of the bowel preparation, the underlying colonic pathology, and the clinical stability of the patient [6]. However, a surgical consultation should be obtained in all cases of perforation [73].

Whenever the risk of a large perforation is present and the patient presents with signs and symptoms of peritonitis, the emergency surgery approach is reasonable and safe [6]. Surgical management is also recommended in patients with concomitant colonic diseases requiring surgery, transplanted patients, and immunosuppressed patients [36, 74]. In selected patients with localized pain, free air without diffuse free fluids in radiographs, hemodynamic stability, and an absence of fever, non-operative management (conservative) may be appropriate [61, 68, 75–78] and is associated with low morbidity, low mortality, and short hospital stays. Conservative management is usually suitable for small, sealed-off perforations that occurred during a therapeutic colonoscopy in patients with an optimal bowel preparation [8, 23, 24].

Conservative treatment consists of serial clinical and imaging monitoring (every 3–6 h) with absolute bowel rest, intravenous fluids for hydration, intravenous administration of broad-spectrum antibiotics, and a close multidisciplinary team follow-up to promptly detect the development of sepsis and peritoneal signs [6, 78, 79]. Drainage of the peritoneal air through a Veress needle punction may be useful in relieving abdominal pain, improving respiratory function, and facilitating the closure of the perforation site [80]. The overall success rate of conservative treatments for colonic perforation varies from 33 to 90% [36].

An early success with non-surgical treatment does not rule out the potential need for surgery [52]. If the conservative treatment is successful, clinical improvement will gradually occur within 24 h, but a continuous and strict clinical and biochemical follow-up is recommended. In cases of clinical deterioration or progression to a septic condition or peritonitis, surgical treatment should not be delayed. The sole presence of subdiaphragmatic free air does not constitute an indication for urgent surgery. Of note, complication rates and lengths of hospital stay are significantly higher in patients who have undergone surgery after conservative management than in patients who were initially treated with surgery [81]. Indeed, when surgical treatment is delayed, the peritonitis and colonic wall inflammation could worsen, requiring a more invasive surgery that is associated with a poorer prognosis [13, 82]. Ideally, the decision to pursue surgery should be made as early as possible after the endoscopy [2].

Endoscopic treatment is possible when the perforation site is recognized intra-procedurally or within 4 h following the procedure and the bowel preparation is still adequate [45]. Urgent endoscopic therapy with clip placement and the use of CO_2_ may limit the volume of extraluminal insufflation and subsequently the need for surgery [83–85]. Endoscopic clip closure of ICP was first reported in the literature in 1997 [86]. Today, it should be considered a valuable non-invasive method for ICP that is recognized during a colonoscopy. It has been shown to be effective in sealing and healing the perforation and avoiding surgery in most cases [2]. The decision to perform the endoscopic closure of colonic perforation depends on the size and the cause of the iatrogenic damage as well as the endoscopist’s experience and the availability of appropriate endoscopic devices [45]. Clipping closure of ICP is recommended for small perforations (less than 1 cm) originating from either diagnostic or therapeutic colonoscopies [2, 24, 87], with a success rate of 59–100% [2, 4, 88, 89]. In larger or difficult perforations, a combination of endoclips and endoloops might be used. There are also few reports in the literature about closure with conventional clips for perforations larger than 1 cm [90–92]. A limitation of the endoscopic closure is the difficulty of evaluating the completeness of the colonic closure after the clip application. This might result in delayed complications such as intra-abdominal abscesses, which can occur due to the persistence of intestinal fluids in the peritoneal cavity or an intermittent leakage [2].

Over the last several years, new devices have been introduced to widen the spectrum of possibilities of performing an endoscopic closure of a gastrointestinal perforation. Through-the-scope (TTS) clips and over-the-scope clips (OTSC) are both effective for the early closure of defects smaller than 2 cm, with overall technical and clinical success rates of 93 and 89%, respectively [88, 93–95]. TTS clips are more suitable for closure of small therapeutic perforations (less than 1 cm), whereas OTSC may be used for larger defects. The OTSC is a nitinol clip shaped to mimic a trap that allows for the inclusion of more tissue and consequently closure of larger perforations than the conventional clips [96]. Recent studies focusing on the outcomes after OTSC placement revealed a rate of procedural success of 80–100% and clinical success rates of 57–100% [96–98].

The overstitch endoscopic suturing device (Apollo Endosurgery, Austin, TX, USA) was recently developed and might play a role in the future ICP closures [99]. Partially or totally covered stenting could potentially allow closing the perforation, but data supporting its clinical application are still lacking. A clear indication for surgery in the setting of endoscopic treatment of an ICP consists of a complicated procedure or a failed endoscopic closure with an ongoing leak that is causing fecal peritonitis [45].

##### Statement 5

5.1. * Non-operative (conservative) management of ICPs may be appropriate in selected patients, including patients who are hemodynamically stable, without sepsis, experiencing localized pain, and with no free fluid in radiographs (Recommendation Grade 1C).*
5.2. * Endoscopic treatment can be considered as an initial approach if it is feasible within 4 h following the procedure depending on the size and cause of the iatrogenic injury and the operator’s level of experience (Recommendation Grade 2C).*
5.3. * Emergency surgery is recommended when the patient develops signs and symptoms of peritonitis, in cases of clinical deterioration, suspected large perforation, failure of conservative management, poor bowel preparation, or in the presence of an underlying colonic disease requiring surgery (Recommendation Grade 1A).*

#### What is the minimum duration of the hospital observation period for patients who have undergone successful endoscopic closure or conservative management of ICP?

After a successful endoscopic closure, it is advisable that a multidisciplinary team, including abdominal surgeons, endoscopists, gastroenterologists, and anesthesiologists, are involved in the patient’s follow-up [52]. Fasting, broad-spectrum antibiotic therapy and intravenous hydration are the basis of treatment [3, 88, 100]. Close observation for signs of peritoneal irritation and monitoring of biochemical inflammatory parameters are crucial. When pain disappears and the inflammatory parameters and bowel function return to normal, oral intake can be resumed [100]. The duration of observation is subjective but obviously related to the patient’s status and the response to the conservative (non-operative) or endoscopic treatment. The mean duration of hospital stay after non-surgical ICP management ranges from 9 to 13 days [88].

##### Statement 6

6.1. * After conservative or endoscopic treatment of ICP, monitoring and follow-up should be assured by a multidisciplinary team, including surgeons. There is no optimal duration of the observation period, but it depends on the patient’s clinical status and response to treatment (Recommendation Grade 1C)*

#### Which investigations (clinical, biochemical, and imaging) should be performed during the observation period in patients who have undergone successful endoscopic closure or conservative management of ICP?

There are no studies in the literature focusing specifically on the clinical and biochemical follow-up of patients who have undergone endoscopic closure or conservative management of ICP.

The available evidence is mainly supported by retrospective series. During the observation period, the patient treated for ICP should be monitored clinically as well as through laboratory values and imaging. Clinically, peritoneal signs such as tenderness, rebound tenderness, and muscle guarding, as well as signs of infection, such as fever, nausea, vomiting, abdominal distension, and diarrhea, should be recorded [36, 69]. Frequent assessment of the physical status and vital signs should be completed by laboratory tests for WBC, CRP, Hb, blood urea nitrogen, PCT, and electrolytes [66]. As an imaging technique, the CT scan remains the most accurate tool to be performed in case of clinical deterioration, especially when the need for surgery is in question and before discharge for non-operative treatments.

##### Statement 7

7.1. * During the observation period, the patient treated for ICP should be monitored clinically, by laboratory tests (including WBC, PCT, CRP) and imaging (CT scan) (Recommendation Grade 2C).*

#### What is the recommended type and duration of antibiotic therapy in patients who have undergone successful endoscopic closure or conservative management of ICP?

In patients who have undergone endoscopic repair of ICP, infection control is usually attained with a short-term course of antibiotic therapy (3–5 days). Antibiotics should be stopped if there are no signs of systemic inflammation and/or peritonitis after the short-term treatment. Considering the composition of the intestinal microbiota in the large bowel, patients with ICP require antimicrobial coverage for Gram-negative bacteria as well as for anaerobes. The potential infecting organisms in colorectal procedures are derived from the bowel lumen, where *Bacteroides fragilis* and other obligate anaerobes as well as *Enterobacteriaceae* such as *Escherichia coli* are the most common bacteria [101]. If there is any sign of an ongoing infectious process, antibiotics should be continued. An abdominal CT is recommended after 5–7 days to exclude residual signs of peritonitis or abscess formation and to exclude the possible need for a surgical intervention.

The duration of antimicrobial therapy in patients with complicated intra-abdominal infections has been debated. Antibiotic therapy should be shortened in those patients demonstrating a positive response to treatment. A prospective trial published recently by Sawyer et al. demonstrated that, in patients with complicated intra-abdominal infections undergoing an adequate source-control procedure, the outcomes after approximately 4 days of fixed-duration antibiotic therapy were similar to those after a longer course of antibiotics that extended until after the resolution of physiological abnormalities [102].

##### Statement 8

8.1. * In patients who have undergone conservative management of ICP, even if there is no sign of diffuse peritonitis, antibiotic therapy covering Gram-negative bacteria and anaerobes is recommended (Recommendation Grade 1C).*
8.2. * In patients with perforation repaired by endoscopic closure, a short-term course of antibiotic therapy (3–5 days) covering Gram-negative bacteria and anaerobes is recommended. Antibiotics should be stopped if there are no signs of systemic inflammation and/or peritonitis after the short-term treatment. Abdominal CT is suggested to help rule out peritonitis or early abscess formation (Recommendation Grade 1C).*
8.3. * In patients who have undergone a surgical procedure with an adequate source-control procedure, postoperative therapy should be shortened as much as possible after the resolution of physiological abnormalities (Recommendation Grade 1C).*

#### Which is the recommended type and duration of antithrombotic prophylaxis in patients who have undergone successful endoscopic closure or conservative management of ICP?

Sepsis is associated with activation of blood coagulation (hypercoagulability) contributing to venous thromboembolism (VTE) [103–105]. Patients with abdominal sepsis may be at increased risk of VTE due to their premorbid conditions, surgical intervention, admitting diagnosis of sepsis, and events and exposures such as central venous catheterization, invasive tests and procedures, and drugs that potentiate immobility. A prospective cohort study using the National Surgical Quality Improvement Program database of the American College of Surgeons (ACS-NSQIP) was designed to evaluate the impact of preoperative sepsis on the risk of postoperative arterial and venous thrombosis. The study included 2,305,380 adults who underwent a range of surgical procedures [106]. The systemic inflammatory response syndrome was defined by the presence of two or more of the following: temperature > 38 or < 36 °C; heart rate > 90 beats/min; respiratory rate > 20 breaths/min or a PaCO_2_ < 32 mmHg (< 4.3 kPa); white blood cell count > 12,000 cells/mm^3^, < 4000 cells/mm^3^, or > 10% immature band forms; or anion gap acidosis (> 12 mEq/L). Among all surgical procedures, patients with preoperative systemic inflammatory response syndrome or any sepsis had three times the odds of having an arterial or venous postoperative thrombosis. The risk of thrombosis increased with the severity of the inflammatory response and was higher in both emergent and elective surgical procedures. Thus, patients with ICP should be considered at risk, and thromboprophylaxis should be recommended.

##### Statement 9

9.1. * In patients with ICP undergoing a surgical procedure, thromboprophylaxis is generally recommended during hospitalization and thereafter according to the underlying disease and comorbidities (Recommendation Grade 1B).*

#### How long is it recommended that patients fast following successful endoscopic closure or conservative treatments for ICP?

There are no prospective clinical trials assessing the necessary duration of fasting following non-operative management or endoscopic repair of ICP. In the setting of conservative treatment, the general recommendations called for “bowel rest,” but the duration is unclear. Retrospective studies reported fasting durations of between 2 and 6 days. In one of the largest series, 24 patients with ICP were managed with conservative treatment, which failed in 3 patients; 31 patients were initially clipped, of which 22 procedures were successful. Poor outcomes were related to patient age, ASA status, and failure of conservative treatment. The only significant predictor of failure of the conservative treatment was the perforation size. Fasting duration did not appear to impact the outcomes [81].

Park et al. [69] reported a single-center series on ICP including 15 patients managed with either conservative treatment (*n* = 4) or endoscopic repair (*n* = 11) and compared these patients with 35 patients managed surgically. The duration of fasting was significantly shorter in the non-surgery group than in the surgery group (3.8 vs. 5.6 days). The mean fasting time was also 1 day shorter for patients treated by endoscopic repair versus surgery in the study by Kim et al. [4]. Moreover, the fasting duration was not related to ICP treatment failure.

It has been suggested that a clear liquid diet can begin immediately after the endoscopic repair of ICP; the evidence is not strong, but there are no data to indicate that this practice is not feasible or unsafe [36]. Following open or laparoscopic repair of ICP, there is no restriction on oral intake, as supported by numerous studies that provided enteral nutrition in the early period after colorectal surgery [107].

##### Statement 10

10.1. * A liquid diet may begin within 1 to 2 days after the initiation of conservative management of ICP, according to the patient’s clinical status (Recommendation Grade 1C)*
10.2. * A liquid diet may begin immediately after endoscopic repair of ICP, according to the patient’s clinical status (Recommendation Grade 1C)*

### Surgical treatment of ICP

#### Is explorative laparoscopy indicated in all patients with ICP?

Surgery is indicated as the first treatment in patients with ongoing sepsis, signs of diffuse peritonitis, large perforations, and failure of conservative management and in the presence of certain concomitant pathologies, such as unresected polyps with high suspicion of being a carcinoma [6, 60, 78].

The peri-operative morbidity and mortality related to surgery for ICP are considerable, with rates of 21–44% and 7–25%, respectively [5, 13–18]. Particularly frail patients, such as older patients and patients with low preoperative blood pressure, can have higher risks of mortality associated with colorectal perforation [108]. Thus, appropriate patient selection and surgical procedures are crucial in limiting the morbidity and mortality related to surgery for ICP.

In general, intraoperative findings determine the best technique to apply according to the different scenarios. Surgical procedures for the management of ICP include colorraphy, wedge resection, colostomy by exteriorization of the perforation, and colonic resection with or without primary anastomosis or stoma. The decision regarding the type of surgical procedure depends on (a) the size, location, and etiology of the ICP; (b) the viability of the surrounding colon and mesocolon; (c) the degree of and time from the development of peritonitis; (d) the patient’s general status and the presence of comorbidities; (e) the quality of the colonic preparation; and (f) the presence of residual lesions not resected during the colonoscopy procedure [2, 8, 24, 60, 82, 109, 110].

The decision of which procedure to perform, therefore, depends on many variables, and it must be made after a careful inspection of the whole colon and peritoneal cavity. Explorative laparoscopy should be considered a minimally invasive technique useful for performing both diagnostic and potentially therapeutic procedures. A timely application of explorative laparoscopy may prevent ongoing inflammation and injury that would necessitate more invasive measures, such as open laparotomy and/or colonic diversion [82]. The use of laparoscopy allows for visualizing the parietal defect and its size and specific location, as well as for identifying the potential cause of the perforation (e.g., perforation caused by the shaft of the endoscope, cautery, presence of mesenteric hematomas, emphysema, or effusions), which, as previously stated, are the main factors influencing the choice of treatment option. Early diagnosis is mandatory, and when timely management is ensured, laparoscopy can be the best option, offering reduced morbidity and length of stay and faster postoperative recovery. If no underlying lesion requiring surgical resection is seen during the endoscopy, the size of the tear is small, and the colon is healthy and well perfused, then a laparoscopic primary repair can be safely performed [52, 111].

Moreover, laparoscopic exploration allows the presence of potential signs of peritonitis to be evaluated and eventually allows aspiration, culture, and irrigation of the peritoneal cavity to be performed. Indeed, peritoneal washout and drainage have gained acceptance in the treatment of more advanced cases of colonic infection, such as Hinchey grade 2–3 diverticulitis [112]. Accordingly, the treatment of less advanced inflammatory processes, such as ICP, seems reasonable and indicated.

To summarize, explorative laparoscopy is indicated:

- For both diagnostic and therapeutic purposes [5, 9, 13, 17, 52, 100, 109, 113–119], and depending on the surgeon’s skills, the potential exists for definitive surgical procedures, including suturing the defect, wedge resection, and segmental resection with or without anastomosis and/or stomia
- In questionable situations to rule out the need for further treatments, including laparotomy [82, 118, 120]
- In the case of failure of endoscopic treatment or an inability to perform endoscopic clip application after visualization of the ICP intra-procedurally
- In the case of development of peritonitis after a defined period of observation following perforation

Explorative laparoscopy has a significantly lower morbidity and mortality compared with explorative laparotomy in the emergency setting [121]: specifically, the reported postoperative complication rate is 18.2% for laparoscopy vs. 53.5% for laparotomy. The postoperative mortality rate is 1.11% for laparoscopy vs. 4.22% for laparotomy; and the need for further procedures is significantly lower for laparoscopy (1.11%) than for laparotomy (8.45%).

Explorative laparoscopy may not be indicated when there is:

- A potential risk for anesthesia-related complications, particularly in elderly or frail patients [122, 123], or any contraindications to surgery in general (e.g., hemodynamic instability, coagulopathy, or associated co-morbidities) [9, 122, 123]
- Recent laparotomy or previous abdominal surgery (more than 4 laparotomies) with extensive adhesions and a high risk of iatrogenic injury (relative contraindication)
- The presence of massive bowel dilatation (relative contraindication)
- Aorto-iliac aneurysmal disease (relative contraindication)

The potential diagnostic/therapeutic value of explorative laparoscopy should also be compared with the role of a CT scan in the evaluation of ICP. There is no study in the literature focusing on whether explorative laparoscopy should be performed instead of CT scans in patients with highly suspected ICP. However, when comparing these two modalities for penetrating abdominal trauma, CT scans have a sensitivity/specificity rate of 95%/95%, whereas explorative laparoscopy can achieve a 67–100% sensitivity and 50–100% specificity [121]. Thus, a CT scan should be performed in all cases before contemplating explorative laparoscopy, with the only obvious impediment being hemodynamic instability.

##### Statement 11

11.1. * Explorative laparoscopy is safe and can be considered as the preferred first-line surgical approach for the management of ICP (Recommendation Grade 1C).*
11.2. * Explorative laparoscopy should be performed according to the surgeon’s experience and skills, as well as the availability of adequate technology and surgical devices (Recommendation Grade 1C).*

#### Which are the indications for conversion from laparoscopy to open surgery in patients with surgical ICP?

Thanks to the improvements in minimally invasive surgery, the laparoscopic approach has been increasingly used in recent years, and it should currently be considered a safe and feasible technique for the management of ICP [9, 24, 82, 113, 124–126]. Current literature comparing outcomes of laparoscopy versus laparotomy for the treatment of ICP is scarce and consists mainly of small retrospective studies. The first relevant study was published in 2008 [110] and compared the perioperative outcomes between laparoscopic and open procedures for ICP by including only primary colonic closures without diversion. The authors found fewer complications and a shorter length of hospital stay for the patients in the laparoscopic group [110]. Other studies by Rothold et al. [125] and Schloricke et al. [127] also observed fewer postoperative complications and significantly shorter hospital stays when utilizing the laparoscopic approach. Similar studies with similar results were published by Coimbra et al. [124] and Kim et al. [128], although in these studies, delayed surgeries (> 24 h) and ostomy formation rates were more frequently observed in the open groups, with higher primary repair rates in the laparoscopic groups.

Due to its favorable short-term outcomes, laparoscopy should be considered the preferred approach for both exploration and repair of ICPs that are not manageable with medical treatments. However, the surgeon’s experience and skills are the key factors limiting the applicability and feasibility of laparoscopic ICP management. Conversion from laparoscopy to laparotomy should be considered whenever necessary. The most frequent reasons for conversion are the inability of the surgeon to complete the procedure laparoscopically, the large size of the ICP defect, the extensive peritoneal contamination, the highly inflammatory or neoplastic conditions of the colon, and the patient’s hemodynamic instability.

##### Statement 12

12.1. * Conversion from laparoscopy to laparotomy should be considered whenever necessary with regard to the ability of the operator to proceed laparoscopically, the tissue viability, and the patient’s status (Recommendation Grade 1C).*

#### What are the key factors upon which to choose the best surgical approach for ICP?

The choice of the surgical approach and technique mainly depends on the underlying pathology (e.g., colon cancer, diverticulitis) and the size of the ICP. Primary surgical repair can be used if the colonic tissue appears healthy and well vascularized and if suturing the perforation edges could be performed without tension [24, 113]. Wedge resection is feasible if it does not imply an excessive narrowing of the colonic lumen (e.g., cecum) [108]. Whenever the perforation is too large, the edges appear devitalized, or an avulsion of the adjacent mesocolon is seen, colonic resection might constitute the best option. Generally, patients who undergo surgery within 24 h are more appropriate candidates for less invasive techniques, such as primary suturing of the defect or linear wedge resection. In cases of delayed surgery (> 24 h from the colonoscopy), extensive peritoneal contamination, important comorbidities, or a deterioration of the general status of the patient (i.e., sepsis), a staged repair or colostomy by exteriorization of the perforation (e.g., double-barreled colostomy) must be considered [36, 52].

Currently, there are no prospective or retrospective studies in the English literature comparing the different types of repair (primary suture or wedge resection vs. segmental resection). Therefore, the choice of the surgical technique appears to be mainly empirical, and it is left to the surgeon’s discretion according to the intraoperative findings. Independent of the surgical approach, the main goal of the therapy is the rapid diagnosis, repair, and prevention of abdominal sepsis. If an ICP is to be repaired laparoscopically, the operating surgeon and the surgical team should be comfortable with the laparoscopic techniques, such as mobilization of the colon and intracorporeal suturing. A clinical algorithm mainly based on the size of the perforation and the necrotic area was proposed in 1999 to assist in choosing which type of repair to perform [8]. The maximal size for sutured repair was set at 1 cm. Between 1 and 2.5 cm, a transverse tangential stapled resection was recommended, whereas above 2.5 cm, a segmental resection was indicated [8, 129]. The condition of the bowel to be repaired and the level of contamination and inflammation are the most important factors in determining whether the laparoscopic approach is safe [109]. Both sutured and stapled repair techniques seem to be safe and feasible to repair defects of up to 4 cm [82].

In case of perforated colon cancer, surgery must follow the oncologic principles of cancer resection.

##### Statement 13

13.1. * The best surgical technique for the management of ICP should be decided after a careful inspection of the abdominal cavity and considering the underlying colonic pathology (Recommendation Grade 2C).*
13.2. * Primary repair can be used if the colonic tissues appear healthy and well vascularized, and an approximation of perforation edges could be done without tension (Recommendation Grade 2C).*
13.3. * Wedge resection would be feasible if it does not imply an excessive narrowing of the colonic lumen (e.g., perforation of the cecum or sigmoid colon) (Recommendation Grade 2C).*
13.4. * Colonic resection may be indicated if the perforation is too large, the edges appear devitalized, or an avulsion of the adjacent mesocolon is seen (Recommendation Grade 2C).*
13.5. * Staged repair or colostomy may be necessary in cases of delayed surgery (> 24 h from the colonoscopy), extensive peritoneal contamination, important comorbidities or a deterioration of the patient’s general status (i.e., hemodynamically unstable or sepsis) (Recommendation Grade 2C).*

#### What are the indications for performing a diverting or terminal stoma in patients with ICP?

The formation of a stoma is often included in the overall surgical strategy for the management of ICP. However, no randomized controlled trials or other high-level evidence trials exist to guide this operative decision in this specific indication. Case series of ICP report variable rates of stoma formation (up to 59.7%) [59, 114, 116, 126, 130]. As such, the formation of a stoma forms an adjunct to the overall treatment strategy for these patients.

The precise clinical or operative reasons for stoma formation are incompletely reported in the case series on ICP. Furthermore, these reports are generally limited by their largely retrospective study designs and low event numbers, complicating subgroup analyses. Notwithstanding these limitations, some authors have established increased stoma formation rates in patients with delayed diagnoses, significant peritonitis, and patients with left-sided perforations [114, 126]. Apart from these observations, the limited publications in this area infer that surgical judgment remains essential in the decision-making surrounding the formation of a stoma. Finally, no data exist to specifically address the type of stoma formation in ICP.

##### Statement 14

14.1. * Stoma formation is an accepted and practiced adjunct in the surgical management of ICP (Recommendation Grade 1C).*
14.2. * Surgical judgment is crucial in the decision regarding stoma need: patient, disease, and situational/environmental factors need to be considered in the individual clinical circumstance (Recommendation Grade 1C).*

#### What are the indications for drainage in patients with ICP?

The placement of an intra-abdominal drainage after surgical management of an ICP can be justified by either the presence of peritoneal contamination or the early diagnosis of a potential bleeding or leakage of the repair used for the perforation (i.e., colorraphy, wedge resection, colonic resection) [131–133]. There are no studies available in the literature focusing on the indications of abdominal drainage after successful surgical treatment of ICP. The decision is left to the discretion of the surgeon according to the ICP setting, the intraoperative findings, the type of surgical procedure performed, the adequateness of infection source control, and the patient’s general status [5, 14, 108].

##### Statement 15

15.1. * In the case of early surgery (< 24 h from colonoscopy) in a patient with good bowel preparation, minimal peritoneal contamination, and adequate infection source control, intra-abdominal drainage placement should be avoided (Recommendation Grade 2C).*
15.2. * In the case of delayed surgery (> 24 h from colonoscopy) in a patient with poor bowel preparation or extensive peritoneal contamination, drainage placement may be recommended (Recommendation Grade 2C).*

#### What are the indications for the use of damage control surgery in patients with ICP?

At present, no study concerning ICP and damage control surgery (DCS) is available in the literature. However, once colonic perforation has occurred, the course of sepsis will develop independent of the underlying disease. Thus, to evaluate the use of DCS in cases of ICP, we could analyze the experience in similar settings, such as in perforated diverticulitis (PD), equating ICP to PD [134, 135].

Damage control is a surgical technique originally used in trauma surgery consisting of three stages: (1) an abbreviated initial laparotomy with the aim of controlling hemorrhage and contamination with temporary abdominal closure (TAC); (2) resuscitation until normal physiology is improved; and (3) return to the operating room after 24–72 h for definitive injury repair and abdominal wall closure [136–138].

Untreated or misdiagnosed ICP can progress to peritonitis and sepsis, resulting in serious morbidity and a very poor prognosis. Notably, morbidity rates as high as 43% and mortality rates as high as 25% have been reported [17, 20, 36, 50, 60, 139]. Nearly one quarter of patients will receive a delayed diagnosis, with a 45% incidence of fecal peritonitis [140]. The resultant inflammatory process associated with peritonitis clearly limits the operative options, precluding a single-stage procedure and resulting in fecal diversion in 38% of patients with fecal peritonitis. Several studies reported that age > 67 years, ASA score, blunt injuries, poor bowel preparation, and steroids are risk factors for increased postoperative morbidity (Table 5) [20, 123, 141, 142].

**Table 5:** Risk factors to evaluate when considering damage control strategy for iatrogenic colonoscopy perforations (ICP)

Risk factors	Description	References
Age	> 67	[140]
Delayed diagnosis	> 24 h	[140, 142]
Hemodynamic instability	Need for vasopressors before or during surgery	[123, 143]
“Blunt” ICP	Perforation caused by excessive dilatation or during diagnostic procedures	[142]
Medication use	Chronic steroid therapy	[13, 140]
Severe sepsis	Peritonitis with organ failure	[135, 141]
High surgical risk	ASA III and IV	[142]

Over the last decade, DCS has become a valuable technique in unstable patients with fecal peritonitis [36, 136, 143]. The potential progression of ICP in fecal peritonitis is as probable as it is in perforated diverticulitis. In accordance with the WSES guidelines for the management of acute left-sided colonic diverticulitis, DCS may be suggested for clinically unstable patients (severe sepsis/septic shock) [135]. Critically ill patients with severe sepsis, hemodynamically unstable patients with hypotension, and patients with myocardial depression combined with coagulopathy are not candidates for endoscopic treatment or immediate complex operative interventions. In such patients, DCS allows rapid source control, enhances physiologic optimization, improves primary anastomosis rates, and decreases the need for stoma formation [144]. Therefore, in patients with abdominal sepsis, the application of DCS is individualized but not routinely used, as suggested by current clinical guidelines [145], stressing the importance of a careful assessment by the surgeons. Clearly, an individual approach tailored to each patient’s clinical status might be the most appropriate. In cases of ICP, DCS should be performed in combination with the resection of the perforated colonic segment to bridge the patient to the definitive injury and colonic continuity repair. DCS can represent a very resource-heavy procedure for institutions, however, because of the requirements for access to facilities (operating rooms and intensive care units) and committed staff.

##### Statement 16

16.1. * DCS following ICP may be indicated in hemodynamically unstable patients, patients receiving a delayed diagnosed of ICP, and patients presenting with significant comorbidities (Recommendation Grade 2C).*
16.2. * DCS can be a valid option in cases of staged procedures, particularly when oncologic resections are required (Recommendation Grade 2C).*

### Follow-up of ICP

#### Is there any recommendation to perform a surveillance endoscopy after successful ICP treatment? If any, what is the recommended timing for it?

At present, there are no studies in the literature focusing on the indications and timing for surveillance endoscopy after successful ICP treatment. However, based on the available evidence and clinical experience, a surveillance colonoscopy may be performed based on the initial indication (e.g., benign or malignant pathology) and type (e.g., screening or interventional) of the primary colonoscopy (during which the ICP occurred) and considering the risk-benefit ratio of performing an endoscopic exam [146, 147].

Colonoscopy is specifically contraindicated in cases of known or suspected perforation [148]. Consequently, any endoscopy after ICP treatment should be performed once the colonic wall has completely healed. Assuming that the healing time after ICP treatment is comparable to that after surgical sutures or anastomosis, a surveillance endoscopy may be indicated after approximately 3 months from the successful ICP treatment, depending on the size of the perforation and the type of repair [149].

In general, prior to any surveillance colonoscopy, it is necessary to carefully re-evaluate the presence of specific conditions favoring perforation, including increasing age, female gender, low BMI, intensive care unit stay, inpatient setting, diverticular disease [150], Crohn’s disease [30], obstruction as an indication for the primary colonoscopy, and invasive interventional colonoscopy [26]. Indeed, colonoscopy is contraindicated whenever the risks for the patient’s health or life are judged to outweigh the most favorable benefits of the procedure [148].

##### Statement 17

17.1. * In cases of perforation occurring during a diagnostic colonoscopy for screening or surveillance of colorectal cancer, a repeat endoscopy is indicated within 3 to 6 months postoperatively if the screening or clearing colonoscopy was incomplete due to malignant obstruction or inadequate preparation (Recommendation Grade 1C).*
17.2. * In cases of perforation occurring during a colonoscopy for gastrointestinal bleeding, a surveillance endoscopy is indicated for diagnostic and therapeutic purposes; in cases of acute lower gastrointestinal bleeding, it is necessary to ascertain the resolution of the perforation (Recommendation Grade 1C).*
17.3. * In cases of perforation occurring during an operative colonoscopy (*e.g.*, polypectomy, endoscopic mucosal resection, or endoscopic submucosal dissection), a surveillance colonoscopy should be performed according to the current guidelines to determine whether the resection during the primary endoscopy was complete. The surveillance endoscopy can be performed within 3 to 6 months from the operative colonoscopy during which the ICP occurred in cases of incomplete resection (Recommendation Grade 1C).*

## Conclusions

**Fig. 2:**
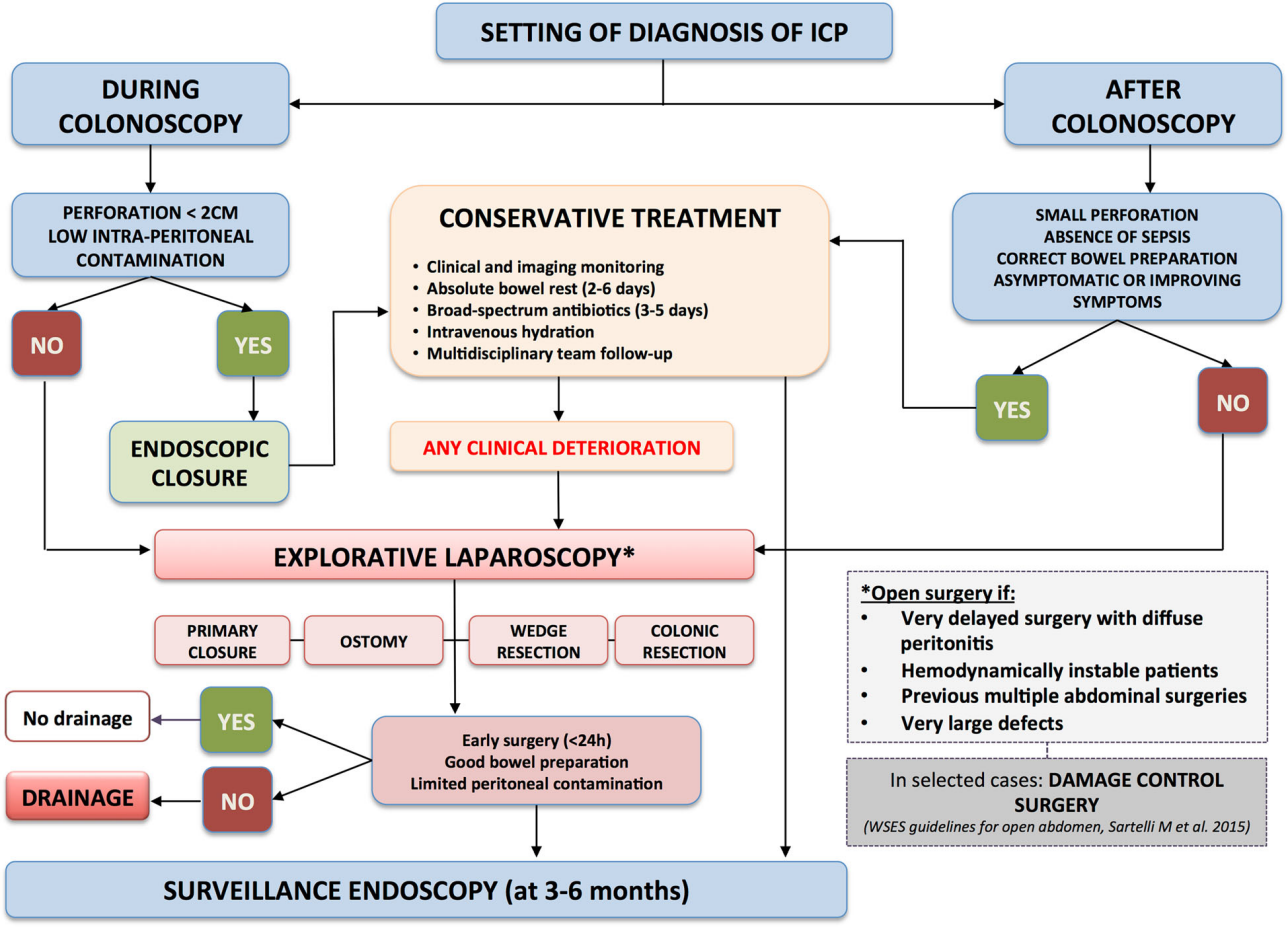
Comprehensive algorithm for the management of iatrogenic colonoscopy perforation

Iatrogenic perforation is a potentially severe complication of colonoscopy that requires a prompt and specific treatment to avoid further morbidity and mortality. In general, a multidisciplinary management, involving gastroenterologists, endoscopists, surgeons, and anesthesiologists, is recommended. The treatment strategy must be chosen based on the clinical setting and the patient’s characteristics, but it should also be adapted to the medical team’s experience and local resources. The comprehensive algorithm presented in Fig. 2 summarizes the management strategies in cases of ICP.

The risk of ICP should be carefully evaluated before a procedure; whenever a risky endoscopy must be performed, the availability of a hospital-based multidisciplinary team can improve patient outcomes. Continuous monitoring and auditing of endoscopic standards and related complications is recommended in each endoscopic center to detect possible performance gaps and improve the safety of colonoscopy. Close collaboration between endoscopists and surgeons is advisable; whenever an ICP occurs, the endoscopist is expected to provide a detailed description of the perforation, procedure, and patient to determine the best treatment option.

Endoscopic repair should be attempted whenever the perforation is detected during the procedure, though outcomes depend on the size and cause of the iatrogenic injury, as well as on the operator’s level of experience.

When the ICP is not immediately detected, it should be suspected and investigated in all patients who present with abdominal pain, tenderness, abdominal distension, fever, and/or rectal bleeding after a diagnostic or therapeutic colonoscopy. CT scan is the most accurate imaging tool to diagnose ICP. Non-operative (conservative) management may be appropriate in selected patients who remain hemodynamically stable in the absence of signs of sepsis. Conservative management consists of complete bowel rest, short-course broad-spectrum antibiotics and intravenous hydration together with close clinical observation.

It must be stressed that early improvement with conservative treatment does not rule out the potential need for surgery. Close monitoring of the patient will allow detection of clinical deterioration, which may signal the need for emergency surgery. Where surgical intervention is required, timely decisions for proceeding with the operation are important. Ideally, these surgeries should occur early and within 24 h of the perforation, as further delays are related to a worse prognosis.

Colonic closure, wedge resection, ostomy, and colonic resection are the main surgical options for ICP management. No RCTs have assessed the superiority of one method over the others. Thus, the therapeutic decision remains essentially empirical, based on the perforation characteristics (e.g., size, time of evolution, and degree of peritoneal contamination), the patient’s general status (e.g., comorbidities), and the availability of adequate technology and surgical devices. Explorative laparoscopy is safe and should be considered the first line approach to assess the perforation-related damages. In patients with good bowel preparation, minimal peritoneal contamination, and adequate infection source control, the perforation repair can possibly be performed by laparoscopy and without drainage placement. Alternatively, staged repair or, in extreme cases, damage control surgery may be required.

The present WSES guidelines contribute to clarifying the complex decision-making process for the management of ICP. Despite the large number of publications, evidence is often derived from observational and moderate to low quality studies. However, it is scarcely feasible to design RCTs for an infrequent complication often requiring emergency treatment. Prospective registries would be highly advantageous to defining the validity of the present recommendations and proposed guidelines.

## Abbreviations

ASA: American Society of Anesthesiologist score
BMI: Body mass index
CRP: C-reactive protein
CT: Computed tomography
DCS: Damage control surgery
EMR: Endoscopic mucosal resection
ESD: Endoscopic submucosal dissection
ICP: Iatrogenic colonoscopy perforation
MDCT: Multidetector computed tomography
OTSC: Over-the-scope clips
PCT: Pro-calcitonin
PD: Perforated diverticulitis
PPV: Positive predictive value
RCT: Randomized controlled trial
TAC: Temporary abdominal closure
TTS: Through-the-scope clips
VTE: Venous thromboembolism
WBC: White blood cell
